# Comparison of Positive End-Expiratory Pressure versus Tidal Volume-Induced Ventilator-Driven Alveolar Recruitment Maneuver in Robotic Prostatectomy: A Randomized Controlled Study

**DOI:** 10.3390/jcm10173921

**Published:** 2021-08-30

**Authors:** Kangha Jung, Sojin Kim, Byung Jun Kim, MiHye Park

**Affiliations:** 1 Department of Anesthesiology and Pain Medicine, CHA Ilsan Medical Center, School of Medicine, Cha University, Pocheon-si 10414, Korea; subjounalid@gmail.com; 2 Department of Anesthesiology and Pain Medicine, Samsung Medical Center, School of Medicine, Sungkyunkwan University, Seoul 06351, Korea; sojin.kim@samsung.com (S.K.); byungjun0613.kim@samsung.com (B.J.K.)

**Keywords:** alveolar recruitment maneuver, atelectasis, electrical impedance tomography, end-expiratory lung impedance, laparoscopy, lung compliance

## Abstract

Background: We evaluated the pulmonary effects of two ventilator-driven alveolar recruitment maneuver (ARM) methods during laparoscopic surgery. Methods: Sixty-four patients undergoing robotic prostatectomy were randomized into two groups: incrementally increasing positive end-expiratory pressure in a stepwise manner (PEEP group) versus tidal volume (V_T_ group). We performed each ARM after induction of anesthesia in the supine position (T1), after pneumoperitoneum in the Trendelenburg position (T2), and after peritoneum desufflation in the supine position (T3). The primary outcome was change in end-expiratory lung impedance (EELI) before and 5 min after ARM at T3, measured by electrical impedance tomography. Results: The PEEP group showed significantly higher increasing EELI 5 min after ARM than the V_T_ group at T1 and T3 (median [IQR] 460 [180,800] vs. 200 [80,315], *p* = 0.002 and 280 [170,420] vs. 95 [55,175], *p* = 0.004, respectively; PEEP group vs. V_T_ group). The PEEP group showed significantly higher lung compliance and lower driving pressure at T1 and T3. However, there was no significant difference in EELI change, lung compliance, or driving pressure after ARM at T2. Conclusions: The ventilator-driven ARM by the increasing PEEP method led to greater improvements in lung compliance at the end of laparoscopic surgery than the increasing V_T_ method.

## 1. Introduction

During general anesthesia, gas exchange is impaired due to a mismatch of the regional distribution of ventilation and perfusion [1]. Furthermore, in laparoscopic surgery, the resultant pneumoperitoneum impairs respiratory mechanics and worsens gas exchange by increasing intra-abdominal pressure and favoring the formation of atelectasis [2]. To facilitate robotic prostatectomy, the patient must be placed in a steep Trendelenburg position combined with capnoperitoneum [3,4]. The main pathogenic mechanism is the development of atelectasis in dorsal-dependent lung areas and over-distension in ventral non-dependent lung areas [5]. Therefore, intraoperative ventilation that recruits collapsed alveoli without causing over-distension of the alveoli may decrease postoperative pulmonary risk by improving perioperative oxygenation and respiratory mechanics and reducing oxidative stress, inflammatory response, and lung injury [6].

The alveolar recruitment maneuver (ARM) is desirable for resolving atelectasis during laparoscopic surgery; however, the potential beneficial effects must be weighed against the potential for harm. Ventilator-driven ARM is a newer technique that mimics manual ARM through modification of the ventilator parameters without disconnection from a ventilator to deliver large tidal volumes via a manual resuscitation bag [7]. During ventilator-driven ARM, stepwise increases in positive end-expiratory pressure (PEEP) or tidal volume (V_T_) may prevent untoward subject responses such as straining or coughing when the depth of anesthesia is equivocal [8,9].

Electrical impedance tomography (EIT) is a non-invasive, radiation-free tool for the assessment of regional lung ventilation at the bedside and in the operating room. It has been demonstrated that EIT reliably and precisely measures the change in lung volume during ARM [9,10]. When compared with electron beam computed tomography (CT), EIT demonstrates a highly significant correlation in measuring regional lung ventilation [11]. The change in end-expiratory lung impedance (EELI) correlates directly with the change in end expiratory lung volume (EELV) [12,13,14,15].

The primary aim of this study was to determine which ventilator-driven ARM method (stepwise increases in PEEP or V_T_) is better in restoring EELV with EELI by EIT monitoring. We hypothesized that the increasing PEEP method would be superior to the increasing V_T_ method in restoring EELV, improving lung compliance and oxygenation after ARM.

## 2. Materials and Methods

### 2.1. Patients and Study Design

This prospective, randomized study was conducted according to the guidelines of the Declaration of Helsinki and approved by our Institutional Review Board (SMC 2019-11-117) and registered with Clinicaltrials.gov (NCT04258202, dates of registration and last update, 6 February 2020 and 10 November 2020). Written informed consent was obtained from all participants. The study was performed from March 2020 to October 2020 at the Samsung Medical Center, Seoul, Korea.

Patients aged between 50 and 75 years with American Society of Anesthesiology (ASA) I-II status undergoing elective robotic prostatectomy were included. Patients with pre-existing lung or cardiac disease, pathologic lung function (pulmonary function test: the ratio of forced expiratory volume in the first second of expiration to forced vital capacity <70% or forced vital capacity <70%), or body mass index (BMI) >35 kg m^−2^ were excluded from the study. Drop-out criteria included interruption of study protocol, bleeding (>500 mL), or severe hypotension (mean blood pressure <55 mm Hg with vasopressor/inotrope) during surgery.

Patients were assessed for eligibility and gave consent on the day before surgery. Randomization was conducted by computer-generated random numbers with a 1:1 ratio, and the allocation was sealed in an opaque envelope (MHP). The sealed envelope was opened just before anesthesia and provided the designated ARM according to the group assignment. Postoperative lung ultrasounds in the PACU were collected by retrieving blinded study logs (KHJ). Downloaded data of EIT and postoperative complications from the medical charts were blindly corrected (BJK). Attending anesthesiologists who were not involved in the study recorded the arterial blood gas analysis and ventilator parameters. A chest X-ray of all patients was taken in the morning on POD 1 and examined by the blinded physician. The group designation was blinded during analysis.

### 2.2. Anesthesia and Surgery

The fraction of inspired oxygen (FIO_2_) was set at 1.0 during preoxygenation and induction. For induction of anesthesia, a 2 mg kg^−1^ propofol bolus with remifentanil continuous infusion was used. Intubation was performed using an endotracheal tube after a bolus injection of 1.0 mg kg^−1^ rocuronium, and a radial arterial catheter was placed for blood sampling and continuous hemodynamic monitoring. Continuous infusion of neuromuscular blockade agent was administered to obtain Train of Four responses between 0 and 1 using neuromuscular monitoring (M-NMT, GE Healthcare, Helsinki, Finland) during ARM.

We used a ventilator (Carestation 650, GE Healthcare, Hatfield, UK) and changes in ventilator settings were made manually. The initial ventilator setting was the volume-controlled mode with a tidal volume of 7 mL kg^−1^ of predicted body weight (PBW), FIO_2_ of 0.60, and PEEP of 5 cm H_2_O. The first ARM was performed 10 min after tracheal intubation and defined as T1. The abdominal cavity was insufflated with CO_2_ to a pressure of 12 mmHg, and the patient was placed in the mild Trendelenburg position after which the trocar cannulas were located at the classical points. Finally, the patients were slowly placed in the steep Trendelenburg position (30° from horizontal). The time of robotic docking in the Trendelenburg position was defined as T2. All operations were performed on the same table with the same degree of Trendelenburg tilt.

The surgeon performed the procedure using the da Vinci Robot Surgical System (Intuitive Surgical, Sunnyvale, CA, USA) using a transperitoneal approach. Intraperitoneal pressure was adjusted by the surgeon as needed (range 12–15 mmHg). At the end of the procedure, the position of the table was neutralized, and the pneumoperitoneum was released and defined as T3. FIO_2_ was increased to 1.0 at 15 min before the planned tracheal extubation. The surgical wounds were closed, and muscle relaxant was reversed with 200 mg sugammadex. Tracheal extubation was performed in the operating room.

In case of ARM interruption due to hemodynamic concerns (heart rate < 45 beats min^−1^ or mean blood pressure < 60 mmHg), 5 mg ephedrine and 100 mL crystalloid were infused. Maintenance crystalloid was infused at a rate of 4 mL kg^−1^ h^−1^.

### 2.3. Study Protocol for Each Recruitment Maneuver

#### 2.3.1. The PEEP Group

The maneuver was initiated with a V_T_ of 7 mL kg^−1^ PBW and an I:E ratio of 1:1, PEEP 5 cm H_2_O, and an increase in the PEEP 5 cm H_2_O every 6 breaths until a peak inspiratory pressure (PIP) of 40 cm H_2_O was reached. Once the target pressure was achieved, the PEEP was maintained for 10 breaths.

#### 2.3.2. The V_T_ Group

The maneuver was initiated with a V_T_ of 7 mL kg^−1^ PBW and an I:E ratio of 1:1, PEEP 5 cm H_2_O, and an increase in the V_T_ by 4 mL kg^−1^ every 6 breaths until a PIP of 40 cm H_2_O was reached. Once the target pressure was achieved, the V_T_ was maintained for 10 breaths.

### 2.4. Measurements

In our study, EIT measurements were performed using the PulmoVista 500 tomograph (Dräger PulmoVista 500, Lübeck, Germany). The electrode belt was placed around the patients’ chest between the fourth and fifth intercostal spaces [16]. EIT uses electrical conductivity of the chest to generate cross-sectional images of the lung inferred from surface electrical measurements realized by a 16-electrode belt. In biological tissue, conductivity varies between tissues depending on air content. The end-expiratory lung impedance variation (ΔEELI) of pre- and post-ARM is expressed as a function of the tidal variation of the respiratory cycle chosen as a reference. We selected four horizontal parallel regions of interest (ROIs) within the chest contour: ROI 1 (ventral), ROI 2 (central ventral), ROI 3 (central dorsal), and ROI 4 (dorsal). To evaluate ventilation distribution, the number calculated per ROI is the sum of impedance changes in this ROI in relation to the sum of impedance changes of the whole EIT image. For instance, a value of 30% in ROI 1 indicates that 30% of the tidal volume variation takes part in this ROI. EIT data were continuously recorded and calculated offline using the Dräger EIT analysis tool, version 6.1 (Dräger Medical) [10].

**Figure 1:**
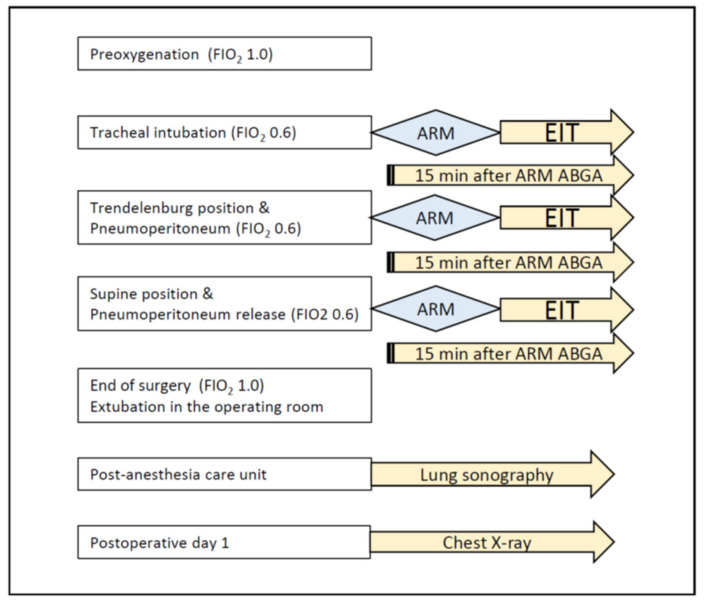
Study protocol. ABGA, arterial blood gas analysis; ARM, alveolar recruitment maneuver; EIT, electrical impedance tomography.

ΔEELI was measured using EIT before ARM, during ARM (maximum hyperinflation), and at 1 and 5 min post-ARM. Static lung compliance (Crs) is calculated by dividing V_T_ by the difference between the plateau pressure (Pplat) and PEEP. Ventilator parameters such as PIP, Pplat, and PEEP were measured before and at 5 min after ARM. Arterial blood gases were measured at 15 min after ARM to assess PaO_2_/FIO_2_ ratio (Figure 1).

Using a Minisono ultrasound system and a linear array 3–12 MHz transducer (Alpinion Medical Systems Co., Ltd., Seoul, Korea). Ultrasonographic assessment of anterior, lateral, and posterior zones (separated by the anterior and posterior axillary lines) was performed, with each divided into upper and lower portions for the right and left lungs. According to the ultrasound pattern, lung ultrasonography score was computed as the presence of the A line alone or fewer than three B lines (0 points), at least three well-spaced B lines (1 point), coalescent B lines (2 points), and lung consolidation (3 points). Global lung ultrasonography score was computed as the sum of the 12 quadrant scores and ranged from 0 to 36 [17].

### 2.5. Statistical Analysis

Our primary aim was to determine which method is better for improving EELI after ARM on T3. Based on a previous study [14], two methods (manual ARM vs. ventilator-driven ARM) of recruitment showed a difference in ΔEELI, with 307 and a standard deviation (SD) of 300, 500. A total of 64 patients was required for a two-sided alpha of 5% and 80% power with 10% dropout or ineligibility.

Categorical variables are reported as the number and percentage. Continuous variables are expressed as the mean ± SD, or median [interquartile]. The normal distribution of data was evaluated with the Kolmogorov–Smirnov test. The primary outcome (ΔEELI) was evaluated with the Mann–Whitney U-test. Demographic data, perioperative data, and secondary outcomes between the two groups were examined with the chi-square test or Fisher’s exact test for categorical variables and independent samples *t*-test or Mann–Whitney U-test for continuous variables. As the three time points were subjected to ARM in the new environment, they were considered independent and no corrections of multiple measurements were adjusted. All of the analyses were performed using SPSS (version 27, Chicago, IL, USA). A two-sided alpha of 0.05 was used for all of the statistical tests.

## 3. Results

**Figure 2:**
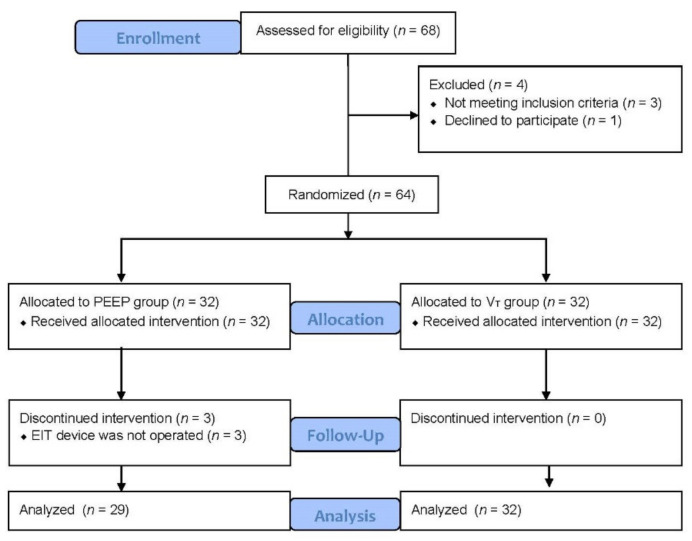
Flow diagram.

A total of 68 patients undergoing elective robotic radical prostatectomy were assessed for eligibility, and 64 patients were entered into the study. Three patients in the PEEP group were excluded due to interruption of the EIT device. Finally, 29 and 32 patients in the PEEP and V_T_ groups, respectively, were analyzed (Figure 2). There were no differences in demographic or operational data between the groups (Table 1). No recruitment maneuver in either group was aborted due to a hemodynamic instability or adverse event.

### 3.1. Effect of the PEEP Group vs. the V_T_ Group on Restoring EELI

**Figure 3:**
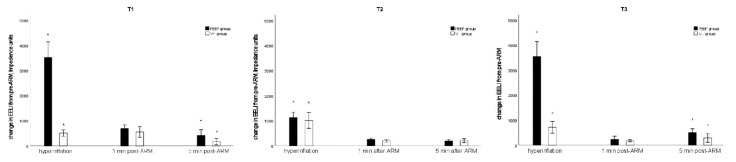
Comparisons of change in end-expiratory lung impedance from baseline during hyperinflation and postalveolar recruitment maneuver at each time point. T1: after induction of anesthesia in the supine position, T2: after pneumoperitoneum in the Trendelenburg position, T3: after peritoneum desufflation in the supine position. * Mann–Whitney U-test for the comparison between the two groups at each time point, *p* < 0.05.

Comparisons of the change in EELI from baseline during hyperinflation and post-ARM at each time point are shown in Figure 3. The primary outcome was the difference in the change in EELI before ARM and 5 min after each ARM at T3 between the two groups. The PEEP group showed a larger increase in EELI at 5 min after each ARM than the V_T_ group (median [IQR] 460 [180,800] vs. 200 [80,315], *p* = 0.002; PEEP group vs. V_T_ group). The PEEP group also showed a larger increase in EELI at T3 than the V_T_ group; however, at T2, the two groups did not show a significant difference in the change in EELI after ARM. When compared with the baseline, both groups showed increased EELI until 5 min after each ARM.

### 3.2. Effect of PEEP Group vs. V_T_ Group on Lung Compliance and Oxygenation

Table 2 presents the comparison of ventilator parameters before and after each ARM at the three time points. Before ARM, patients in both groups were not different in respiratory mechanism condition at all time points. After performing each ARM, the PEEP group showed significantly higher Crs and lower driving pressure at T1 and T3 than the V_T_ group. The change in Crs was significantly greater in the PEEP group than in the V_T_ group at T1 and T3 (mean (SD) 15.8 (10.4) mL cmH_2_O^−1^ vs. 8.3 (6.4) mL cmH_2_O^−1^, *p* = 0.001 and 22.6 (9.0) mL cmH_2_O^−1^ vs. 14.4 (7.3) mL cmH_2_O^−1^, *p* = 0.000, respectively; PEEP group vs. V_T_ group).

At T2, all outcomes showed no significant difference before or after ARM between the two groups. The improvement of Crs after each ARM was small in both groups and did not significantly differ between the two groups (mean (SD) 2.7 (2.1) mL cmH_2_O^−1^ vs. 2.0 (4.3) mL cmH_2_O^−1^, *p* = 0.431; PEEP group vs. V_T_ group).

Oxygenation by PaO_2_/FIO_2_ was not significantly different between the two groups at all three time points. The incidence of inotropic administration was not different between the two groups at all three time points (T: 2 (7%) vs. 0 (0%), *p* = 0.222, T2: 1 (3%) vs. 1 (3%), *p* = 0.475, T3: 3 (10%) vs. 3 (9%), *p* = 0.107; PEEP group vs. V_T_ group). All events were recovered after administration of the first medication dose.

### 3.3. Postoperative Outcomes

There was no difference in lung ultrasound exams in the postanesthesia care unit (PACU) between the two groups (median [IQR] 3 [2,5] vs. 3 [2,4.5], *p* = 0.896; PEEP group vs. V_T_ group). The need for rescue oxygen therapy and atelectasis defined by chest X-ray of postoperative day (POD) 1 was not significantly different (11 (38%) vs. 15 (47%), *p* = 0.481 and 6 (21%) vs. 3 (10%), *p* = 0.514, respectively; PEEP group vs. V_T_ group). There was no difference in the duration of hospitalization between the two groups (median [IQR] 8 [7,8] vs. 8 [7,8.5], *p* = 0.792; PEEP group vs. V_T_ group).

## 4. Discussion

This study compared the effects of two ventilator-driven ARM methods on lung compliance when undergoing robotic prostatectomy. The main findings were that ARM through increasing PEEP in a stepwise manner produced a larger increase in EELI and lung compliance, and a reduced driving pressure in the supine position without pneumoperitoneum relative to increasing V_T_ in a stepwise manner.

ARMs are beneficial in reopening collapsed alveoli and improving lung mechanics, suggesting that performing an ARM after intubation can resolve anesthesia-induced functional residual capacity (FRC) changes [18,19]. However, high-quality supportive evidence is lacking to recommend a routine ARM for all patients during anesthesia [20]. Instead, an ARM may be considered according to an individual risk–benefit assessment.

Consistent with previous results [5], pulmonary compliance decreased from more than 50% of lung compliance before ARM during Trendelenburg positioning and CO_2_ insufflation. After reinstitution of the supine position, lung compliance did not fully return to baseline levels after desufflation following laparoscopy. This is probably caused by basal atelectasis, residual cephalad displacement of the diaphragm, and restriction in diaphragmatic mobility. Instead, a return to baseline levels of lung compliance, driving pressure, and oxygenation can be fully restored using ARM in our study. A previous study also showed restoration of pulmonary compliance after laparoscopic surgery after ARM [21]. These results support that ARM is a necessary component of low tidal volume lung-protective ventilation during laparoscopic surgery.

Conventional manual inflations up to a PIP of 40 cmH_2_O for ARM utilized single sustained manual inflations for 15 s. Manual sustained hyperinflations require a short amount of time to perform, but cause subject responses such as coughing or straining during surgery. During the study period, there were no cases of interrupted ARM due to coughing/straining or hemodynamic instability. A previous study showed manual ARM was superior to ventilator-driven ARM in improving lung compliance but it decreased rapidly due to the disconnection from the ventilator circuit; therefore, PEEP was not maintained [22]. Ventilator disconnection causes potential adverse outcomes including loss of EELV, deoxygenation, shear stress of alveoli, inaccuracy of airway pressure, inspiratory flow, and tidal volume. We found the improvement in lung compliance was maintained 5 min after the application of both techniques.

In our study, lung compliance was significantly improved in the PEEP group compared to the V_T_ group. Under general anesthesia, PEEP prevents end-expiratory airway closure due to decreased FRC below the closing capacity in the dependent lung segments and therefore helps airways to remain open [23]. Thus, PEEP is regarded as an anti-derecruitment strategy. However, tidal inflation might result in the opening and closing of distal lung units; thus, the distal airway was collapsed during the end-expiratory period. The PEEP group generated a much greater EELI during hyperinflation. Lung recruitment is basically an inspiratory phenomenon occurring during tidal ventilation, whereas PEEP prevents expiratory derecruitment [24]. As the EELI was measured during the end-expiratory time, the EELI value from EIT might be significantly larger in the PEEP group during maximum hyperinflation.

Despite achieving better EELI with the PEEP group compared to the V_T_ group during surgery, we did not observe prolonged effects on oxygenation, lung ultrasound in the PACU, and chest X-ray on POD 1. After opening up the lungs with ARM, a sufficient level of PEEP may be required to keep the lungs free of collapse and maintain distension of the airways. Additionally, we thought that even after effective ARM, normal alveoli filled with 100% oxygen have a rapid tendency to collapse to form shunts [25]. The resorption atelectasis might be attenuated with an ARM performed with FIO_2_ < 1.0, followed by individual adequate PEEP after ARM. In patients with previously healthy lungs, lung over-inflation following mechanical ventilation with PEEP was found exclusively in nondependent and caudal lung regions [26]. Overinflation of nondependent regions might result in over-distension of already expanded alveoli, reduced perfusion, and an increase in alveolar dead space [18].

We observed that both methods resulted in a small increase in EELI during surgery in the Trendelenburg position with capnoperitoneum. This indicates that increasing PEEP or V_T_ until PIP reaches 40 cmH_2_O could not overcome the high intra-abdominal pressure. Thus, increasing PIP or a longer recruiting time during the capnoperitoneum period may be required to resist intra-abdominal pressure and push the diaphragm for recruiting collapsed alveoli. Another possible consideration is a cranial shift of the diaphragm caused by excessive intra-abdominal pressure, resulting in impedance changes in the abdomen and the principle of EIT. A position close to the diaphragm may lead to misinterpreted measurements [16]. Thus, a significant adverse impact could be seen on EIT images of the thorax when acquired during capnoperitoneum and the Trendelenburg position.

There were several limitations to the present study. Our study has limitations for generalizability. The data were limited to highly selected male and relatively healthy patients undergoing specific surgery. EIT is a validated tool to measure changes in lung expansion, but CT remains the gold standard for the measurement of atelectasis. However, when we considered radiation exposure, time, and cost, CT was precluded. To measure changes in EELV, nitrogen wash-out might be an alternative method [27]. However, this measurement is technically challenging and prone to error intraoperatively. EIT monitoring during surgery had several limitations because EIT measured electrical impedance, so electrical surgical procedures with electrocoagulant were interrupted by EIT monitoring. Additionally, EIT might affect surgical procedure and position; however, accurate measurement of changes in lung volume using EIT is due to a strong linear relationship between the change in electrical impedance and the change in lung volume. We applied a uniform PEEP of 5 cm H_2_O to all patients, not an individualized PEEP.

During our study, two methods of ventilator-driven ARM were performed without adverse events such as movement or coughing during surgery while maintaining hemodynamic stability. Incrementally increasing PEEP ARM showed more beneficial effects than increasing V_T_ ARM on improving lung compliance in the supine position without pneumoperitoneum. At the end of surgery, lung compliance and driving pressure were fully recovered in preoperative status after PEEP ARM. However, both methods were not effective in the Trendelenburg position during laparoscopic surgery. The optimal ARM strategies for determining recruiting pressure and its duration require investigation in various surgical environments and with differing patient characteristics.

## Figures and Tables

**Table 1:** Patients’ characteristics.

	PEEP Group (*n* = 29)	V_T_ Group (*n* = 32)	*p* Value
Age, yr (range)	67 (56, 75)	65 (54, 75)	0.244
Body mass index, kg m^−2^	24.4 (2.5)	25.0 (2.3)	0.323
Height, cm	167 (6)	168 (6)	0.813
Weight, kg	68 (9)	70 (9)	0.360
ASA I/II	2/27	4/48	0.674
Left ventricle ejection fraction, %	65 (4)	65 (5)	0.771
Comorbid conditions			
Hypertension	13 (45%)	14 (44%)	0.933
Diabetics mellitus	9 (31%)	3 (9%)	0.034
Angina	2 (7%)	0 (0%)	0.214
Current smoking	4 (14%)	2 (6%)	0.411
Pulmonary function test			0.401
Normal	19 (66%)	23 (72%)	
Mild obstructive pattern	6 (21%)	4 (13%)	
Duration of anesthesia, min	214 (46)	213 (49)	0.910
Duration of surgery, min	178 (45)	173 (47)	0.705
Duration of capnoperitoneum, min	152 (53)	152 (47)	0.991

Data are shown as number (%) or mean (SD). ASA: American Society of Anesthesiology.

**Table 2:** Comparisons of respiratory mechanics and oxygenation between the two groups at each time point.

	Before Alveolar Recruitment Maneuver			After Alveolar Recruitment Maneuver		
	PEEP Group	V_T_ Group	*p* Value	PEEP Group	V_T_ Group	*p* Value
T1						
ROI (1 and 2), %	57 [48,66]	58 [53,62]	0.729	56 [48,67]	59 [52,62]	0.939
ROI (3 and 4), %	37 [28,42]	36 [31,42]	0.892	38 [27,45]	37 [32,42]	0.746
PIP	15.5 (1.5)	15.4 (1.6)	0.858	14.4 (1.5)	14.5 (1.5)	0.824
DP, cmH2O	7 [6,8]	7 [6,8]	0.829	5 [5,7]	6 [6,7]	0.043
Crs, mL cmH_2_O^−1^	65.8 (12.9)	64.6 (11.1)	0.694	81.7 (14.1)	72.9 (12.0)	0.011
PaO_2_/FIO_2_, mmHg				432.4 (92.9)	395.2 (140.8)	0.245
T2						
ROI (1 and 2), %	57 [48,66]	58 [53,62]	0.405	56 [48,67]	59 [52,62]	0.405
ROI (3 and 4), %	37 [28,42]	36 [31,42]	0.789	38 [27,45]	37 [32,42]	0.451
PIP	26.8 (2.6)	26.9 (3.2)	0.845	25.2 (2.7)	25.6 (3.2)	0.660
DP, cmH_2_O	19 [17,22]	18 [17,21]	0.597	18 [16,20]	17 [16,19]	0.372
Crs, mL cmH_2_O^−1^	23.4 (3.3)	24.3 (3.9)	0.322	26.1 (3.9)	26.3 (5.3)	0.842
PaO_2_/FIO_2_, mmHg				377.2 (126.1)	409.4 (93.2)	0.266
T3						
ROI (1 and 2)	57 [47,61]	56 [49,65]	0.533	56 [45,62]	60 [51,69]	0.183
ROI (3 and 4)	39 [33,45]	38 [29,46]	0.076	42 [33,50]	35 [20,39]	0.462
PIP	16.7 (1.4)	16.8 (1.9)	0.845	15.2 (1.3)	15.5 (1.6)	0.660
DP, cmH_2_O	9 [8,9]	9 [7,10]	0.768	6 [5,7]	6 [6,8]	0.047
Crs, mL cmH_2_O^−1^	55.1 (10.6)	55.9 (9.6)	0.772	77.7 (13.3)	70.3 (12.1)	0.000
PaO_2_/FIO_2_, mmHg				417.0 (102.5)	421.3 (81.4)	0.857

Data are shown as median [IQR] or as mean (SD). Crs; static lung compliance, DP; driving pressure, PIP; peak inspiratory pressure; regions of interest (ROIs) within the chest contour: ROI 1 (ventral), ROI 2 (central ventral), ROI 3 (central dorsal), and ROI 4 (dorsal).

## Data Availability

The data presented in this study are available on request from the corresponding author. The data are not publicly available due to data privacy protection.
